# HIV destigmatization: perspectives of people living with HIV in the Kumasi Metropolis in Ghana

**DOI:** 10.3389/frph.2023.1169216

**Published:** 2023-09-20

**Authors:** Elizabeth Armstrong-Mensah, Emmanuel Ofori, Ernest Alema-Mensah, Thomas Agyarko-Poku

**Affiliations:** ^1^ Department of Health Policy & Behavioral Sciences, School of Public Health, Georgia State University, Atlanta, GA, United States; ^2^ Department of Medicine, Komfo Anokye Teaching Hospital, Kumasi, Ghana; ^3^ Morehouse School of Medicine, Atlanta, Georgia; ^4^ Suntreso Government Hospital, Kumasi, Ghana

**Keywords:** HIV, HIV-related stigma, people living with HIV, HIV destigmatization, Ghana

## Abstract

**Background:**

Human immunodeficiency virus (HIV)-related stigma has been identified as one of the principal factors that undermines HIV prevention efforts and the quality of life of people living with HIV (PLWH) in many developing countries including Ghana. While studies have been conducted on HIV-related stigma reduction, very few have sought the views of PLWH on how this might be done. The purpose of the study was to (i) identify factors that cause HIV-related stigma in Ghana from the perspective of PLWH, (ii) identify challenges that HIV-related stigma poses to the treatment and care of PLWH, and (iii) to obtain recommendations from PLWH on what they think various groups (community members, health care providers, and adolescents) including themselves should do to help reduce HIV-related stigma in Ghana.

**Methods:**

A mixed methods cross-sectional study design was used to collect data from 404 PLWH at the Suntreso Government Hospital in the Kumasi Metropolis of Ghana across six domains using Qualtrics from November 1–30, 2022. Quantitative data was analyzed using the Statistical Package for Social Sciences (SPSS) version 26 and the Statistical Analysis System (SAS) version 9.4. Qualitative data was analyzed using a thematic approach.

**Results:**

Most of the **s**tudy participants (70.5%) said HIV-related stigma in Ghana is due to ignorance. Of this population, 90.6% indicated that they had experienced stigma because they have HIV, causing them to feel depressed (2.5%), ashamed (2.2%), and hurt (3.0%). Study participants (92.8%) indicated that the challenges associated with HIV-related stigma has affected their treatment and care-seeking behaviors. Recommendations provided by study participants for HIV destigmatization include the need for PLWH not to disclose their status (cited 94 times), community members to educate themselves about HIV (96.5%), health care providers to identify their stigmatizing behaviors (95.3%), health care providers to avoid discriminating against PLWH (96.0%), and the need for adolescents to be educated on HIV and how it is transmitted (97.0%).

**Conclusion:**

It is important for the government and HIV prevention agencies in Ghana to target and address co-occurring HIV-related stigma sources at various levels of intersection simultaneously This will help to shift harmful attitudes and behaviors that compromise the health and wellbeing of PLWH effectively.

## 1. Introduction

The human immunodeficiency virus (HIV) is a major threat to the health and quality of life of populations globally. Emerging in the 1980s, HIV attacks, destroys, and weakens the body's immune system against infections (1), and puts infected persons at risk for opportunistic infections such as tuberculosis, severe bacterial infections, and some cancers that their bodies would normally have been able to fend off (2). In 2022, about 38.4 million people were living with HIV (PLWH), 1.5 million had acquired new HIV infections, and 650,000 had died from HIV-related causes globally (3). In that same year, sub-Saharan Africa, which accounts for 15% of the global population (4), had 28 million PLWH, 425,100 AIDS-related deaths, and 874,000 new HIV infections (5), with Ghana, a country in sub-Saharan Africa, accounting for about 345,599 PLWH, 9,859 deaths from AIDS-related causes, and about 16,938 new HIV infections (6, 7). It was in the bid to address the HIV epidemic that the global community launched the Millennium Development Goals (MDGs) in 2000, and the subsequent Sustainable Development Goals (SDGs) in 2015, with their respective targets of reversing HIV by 2015 (MDG 6) and eliminating HIV by 2030 (SDG 3) (8, 9). Owing to these global efforts, public health interventions and campaigns, scientific advances, and technologies such as HIV testing, voluntary counselling and testing (VCT), and antiretroviral therapy (ART), have been developed and implemented across the globe to control and prevent HIV (10). In spite of these efforts, HIV prevention continues to be a global health issue, thanks to the stigma associated with the disease.

Ghana is a country in West Africa located on the Gulf of Guinea. It is bounded to the West by Côte d'Ivoire, the East by Togo, and the North by Burkina Faso. Ghana covers an area of about 238,553 km^2^ and has a population of about 33 million people (11) living in 16 administrative regions and 216 districts (12). In 2020, the overall HIV prevalence rate in Ghana was 1.6% with regional variation (7). Kumasi, a district in the Ashanti region and Accra, a district in the Greater Accra region of Ghana, were reported to have largest numbers of PLWH (7), with the Ashanti region accounting for 76, 672 of that population. Some reasons cited for HIV prevalence in Kumasi are low condom use, high female sex worker activities, infected people not beginning treatment due to the fear of stigma, risky sexual behavior, increased commercial activity, concealment of HIV status among couples, and low socio-economic status (13). To stem the HIV tide, the government of Ghana has expanded access to treatment and care, and is implementing national as well as international programs such as the World Health Organization's TREAT ALL policy (14). This notwithstanding, the stigma related to HIV continues to derail established prevention, treatment, and care efforts.

HIV-related stigma is the prejudice, negative beliefs, feelings, and attitudes towards PLWH, their families, and those who take care of them (15). It is a multidimensional social construct that is not only shaped by individual perceptions and interpretations of microlevel interactions but also by social and economic forces (16). HIV-related stigma significantly impacts the life experiences of individuals both infected and affected by the disease. It manifests in various forms including discrimination, avoidance behavior (refusal to share food or sit by), social rejection (shunning by family members, peers, and the wider community), the erosion of rights, psychological damage, labeling of people as “socially unacceptable”, and the perpetration of physical violence (16). HIV-related stigma may be external or internal (17). External stigma is the actual experience of discrimination, while internal stigma (felt or imagined stigma) is the shame associated with HIV and PLWHs’ fear of being discriminated against (17–19). Internal stigma is a survival mechanism that protects PLWH from external stigma and often results in thoughts or behaviors such as the refusal or reluctance to disclose a positive HIV status, denial of HIV, and an unwillingness to accept help (18, 20–22).

In Ghana, HIV is a disease associated with certain key populations (men who have sex with men (MSM), people who inject drugs, and sex workers). The notion that the disease is solely transmitted through sex and is the consequence of weak morals, sexual promiscuity and personal irresponsibility that deserves to be punished, have contributed to the stigma and discrimination associated with the disease (15). Thus, HIV-related stigma has driven PLWH and key populations to the margins of society where the fear of gossip, verbal abuse, rejection or even violence, makes getting tested, disclosing one's HIV status, or accessing HIV treatment and care very difficult (23).

HIV-related stigma is not only associated with whether a person is living with the disease or not, but also their gender identity, sexual orientation, engagement in sex work, place of treatment, who their health care provider is, and the communities in which they live (24). Therefore, to successfully reduce HIV-related stigma in Ghana, it is important to target and address co-occurring HIV-related stigma sources at various levels of intersection simultaneously as well as among PLWH (24). While studies have been conducted on HIV-related stigma reduction, very few have sought the views of PLWH on how this might be achieved. The purpose of the study was to (i) identify factors that cause HIV-related stigma in Ghana from the perspective of PLWH, (ii) identify challenges that HIV-related stigma poses to the treatment and care of PLWH, and (iii) to obtain recommendations from PLWH on what they think various groups (community members, health care providers, and adolescents) including themselves, should do to help reduce HIV-related stigma in Ghana. The results from the study have been used to inform content and strategies for interventions to reduce HIV-related stigma and discrimination in Ghana.

## 2. Materials and methods

### 2.1. Setting and population

The study was conducted at the Suntreso Government Hospital situated in the Kumasi Metropolis in the Ashanti region of Ghana. The hospital is a leading provider of quality community-oriented health care including pediatrics, disease control, HIV, obstetrics, gynecology, and surgery. It is also the home of several sexually transmitted infection and HIV clinics with over 9,000 patients.

### 2.2. Sampling and data collection

We conducted a cross-sectional, mixed methods study of PLWH who utilize HIV services at the Suntreso Government Hospital. Using convenient sampling, data was collected over a period of four weeks in October 2022 on study participants perceptions of the causes of HIV-related stigma in Ghana, the challenges HIV-related stigma poses to their treatment and care, and recommendations for destigmatizing HIV-related stigma in Ghana. Study participants were privately informed about the study at the HIV clinic and pharmacy waiting areas where they go to collect their medication. PLWH who volunteered to participate in the study were invited to a private room by the pharmacy where they met with a research assistant who explained the purpose and nature of the study and emphasized the voluntary nature of the study before data collection commenced. Study participants completed a one-time six-minute online survey created in Qualtrics. They gave their consent to participate in the study by completing the survey. No personally identifiable information was collected. Quantitative and qualitative data were collected daily.

### 2.3. Ethical approval

Approval for the study was obtained from the Kwame Nkrumah University of Science and Technology in Kumasi, Ghana.

### 2.4. Variables and measurement

The study questionnaire comprised 18 quantitative and qualitative questions across six domains—(i) demographic information, (ii) causes of HIV-related Stigma in Ghana, (iii) individual stigma, (iv) interpersonal stigma, (v) challenges of HIV-related stigma for HIV treatment and care, and (vi) recommendations for HIV destigmatization in Ghana. The demographics variable focused on the sex, age, and educational level of participants. The causes of HIV-related stigma in Ghana variable focused on what study participants believe are the causes of HIV-related stigma in Ghana. The individual and interpersonal stigma variables focused on study participants personal experience of HIV-related stigma, how they felt because of the experience, and the outcomes of the experience. The challenges of HIV-related stigma for HIV treatment and care variable focused on the effects of the stigma experienced on study participant treatment and care in the past 12 months. The recommendations for HIV destigmatization variable focused on what study participants believe will help to reduce HIV-related stigma in Ghana as well as what should be done at the community, care provider, adolescent, caregivers, sex workers and MSM levels to reduce HIV-related stigma in Ghana.

### 2.5. Statistical analysis

Data collected was cleaned and exported from Qualtrics to the Statistical Package for the Social Sciences (SPSS) software version 26 and the Statistical Analysis System version 9.4 for quantitative analysis. Missing quantitative data were excluded from calculations. Qualitative data was manually extracted from the SPSS database and analyzed using a thematic approach. Analyzed qualitative data are presented verbatim (in italics) to convey exactly what study participants said in explanation to certain questions.

## 3. Results

### 3.1. Univariate analysis

#### 3.1.1. Demographic information

A total of 404 PLWH who utilize health services at the Suntreso Government hospital in Kumasi participated in the survey. Majority were female (64.9%), and more than half (60.8%) were 41 years old and above. Over a third of the study participants (37.4%) had completed junior high school, and a few had completed either senior high school (17.3%), vocational school (7.7%) or any formal education (9.4%) (Table 1).

**Table 1:** Descriptive characteristics of participants by Six identified domains.

Variables	Sample size, *n*	Percentage, %
1. Demographics		
Sex		
Male	141	35.1
Female	261	64.9
Total	402	100.0
Age		
18–24	11	2.7
25–40	147	36.5
40+	245	60.8
Total	403	100.0
Educational level		
No school	38	9.4
Primary school	100	24.8
Junior high school	151	37.4
Senior high school	70	17.3
University	13	3.2
Vocational/technical school	31	7.7
Other	1	0.2
Total	404	100.0
2. Causes of HIV-related stigma in Ghana		
What are the causes of HIV-related stigma in Ghana (Multiple)		
Ignorance	285	70.5
Belief that only certain groups of people can get HIV	78	19.3
Fear of contracting HIV	125	30.9
Making negative comments about people who are tested for HIV	282	70.0
The perception that people deserve to get HIV because of their choices	98	24.3
3. Individual stigma		
Ever experienced stigma		
Yes	27	6.7
No	366	90.6
I do not know	11	2.7
Total	404	100.0
What you felt because of the stigma associated with HIV (Multiple)		
Fear	3	0.7
Depression	10	2.5
Isolation	5	1.2
Shame	9	2.2
Worried about discrimination	4	1.0
Hurt	12	3.0
Guilty	2	0.5
None	57	14.1
I do not know	132	32.7
Other		
4. Interpersonal stigma (Multiple)		
Which have experienced because you have HIV		
I have lost friends	12	3.0
People avoid touching me	12	3.0
People I care about have stopped calling me	2	0.5
People are afraid of me	12	3.0
People do not want me around their children	7	1.7
None	377	93.3
5. Challenges of HIV-related stigma for treatment and care		
Sigma related to HIV has affected your treatment and care in the past 12 months		
Yes	375	92.8
No	9	2.2
I do not know	20	5.0
Total	404	100.0
Which have you done because of HIV-related stigma in the past 12 months (Multiple)		
Not taking my medicine	5	1.2
Not staying in care	8	2.0
Unwilling to disclose my HIV/AIDS status	398	98.5
Other	2	0.5
6. Recommendations for HIV destigmatization in Ghana		
What should be done to do away with HIV related stigma in Ghana (Multiple)		
Put a person before their health status	203	50.2
Use non-judgmental language to describe PLWH	365	90.3
People should feel free to talk openly about HIV	305	75.5
Educate the public	396	98.0
What should be done at the community level to reduce HIV related stigma in Ghana (Multiple)		
Thank people living with HIV for disclosing their status	262	64.9
Ask people living with HIV how they can be supported	386	95.5
Reassure people living with HIV that the disclosure of their status will not change your relationship	385	95.3
Educate oneself about HIV	390	96.5
Avoid being judgmental	380	94.1
Treat people living with HIV with dignity and respect	387	95.8
Do not deny people with HIV employment	384	95.0
Stop blaming people living with HIV for getting the disease	386	95.5
Provide quality care to people living with HIV	379	93.8
What should health care providers do to reduce HIV stigma in Ghana? (Multiple)		
Participate in mandatory training on HIV-related stigma led by people living with HIV	382	94.6
Be empathetic towards people living with HIV during treatment and care	386	95.5
Participate in training on HIV transmission, prevention, and care	387	95.8
Identify behaviors that are stigmatizing	385	95.3
Avoid showing occupational fear of acquiring HIV	381	94.3
Provide training in HIV to non-HIV specialty doctors	385	95.3
Make HIV stigma a priority in HIV care and treatment	383	94.8
Create and enforce policies regarding HIV-related stigma	383	94.8
Avoid discriminating against HIV patients	388	96.0
Provide quality care to people living with HIV	380	94.1
What do you think should be done to reduce HIV related stigma among adolescents without HIV (Multiple)		
Educate adolescents on HIV and how it is transmitted	392	97.0
Educate adolescents on the negative effects of HIV-related stigma	391	96.8
Encourage adolescents to talk freely about HIV	375	92.8
Encourage adolescents not to shun people living with HIV	386	95.5
Encourage adolescents not to talk negatively about people living with HIV	386	95.5
What do you think should be done to reduce HIV related stigma among adolescents living with HIV (Multiple)		
Stop discrimination against adolescents living with HIV	390	96.5
Remind adolescents living with HIV to take their HIV medications	388	96.0
Educate schoolteachers and staff about HIV and how it is transmitted	389	96.3
Educate students about HIV and how it is transmitted	386	95.5
Boarding schools should feed HIV students on medication when they need to eat	381	94.3
Encourage adolescents living with HIV to take their HIV medications	385	95.3
Have flexible school schedule for adolescents living with HIV so students can attend clinical appointments	381	94.3
What do you think should be done to reduce HIV related stigma among caregivers (Multiple)		
Do not shun HIV caregivers	370	91.6
Do not make caregivers feel guilty for taking care of someone living with HIV	390	96.5
Do not talk negatively about HIV caregivers	392	97.0
What do you think should be done to reduce HIV related stigma among sex workers (Multiple)		
Sex work should not be criminalized	136	33.7
Avoid talking negatively about sex workers	360	89.1
Avoid making fun of sex workers when they go to seek HIV care and treatment	325	80.4
What do you think should be done to reduce HIV related stigma among MSM (Multiple)		
People should not gossip about MSM	93	23.0
Health care providers should not deny MSM HIV treatment and care	329	81.4
Health care providers should treat MSM with respect when they seek care	148	36.6

#### 3.1.2. Causes of HIV-related stigma in Ghana

In response to what causes of HIV-related stigma in Ghana, the study participants said ignorance (70.5%), the belief that only certain groups of people can get HIV (9.3%), the fear of contracting HIV (30%) and negative comments made about people who get tested for HIV (70%). In addition, some study participants stated that people deserve to have HIV because of their choices (24.3%) and others (5.6%) said they did not know (Table 1).

#### 3.1.3. Individual and interpersonal stigma

Regarding individual stigma, 90.6% of the study participants acknowledged ever experiencing stigma because they have HIV. This, they said, made them feel depressed (2.5%), shame (2.2%), hurt (3.0%), and worried about discrimination (1.0%). A few of the study participants (14.1%) said that they were not bothered about HIV-related stigma or felt nothing, and a third (32.7%) stated that they did not know. When it came to what they have experienced as a result of the stigma related to HIV, 3.0% of the study participants respectively indicated that they have lost friends, people are afraid of them, and people avoid touching them (Table 1).

#### 3.1.4. Challenges of HIV-related stigma for treatment and care

When asked about whether HIV-related stigma has affected their treatment and care in the past 12 months, 92.8% of the study participants said yes, 2.2% said no, and 5.0% said they did not know. Those who said yes said it was because they felt ashamed (cited 2 times) and did not want to be seen at the health facility (cited 2 times). On what they had done because of HIV-related stigma they experienced in the past 12 months, many of the participants (98.5%) stated that they were unwilling to disclose their HIV status, 1.2% said they had stopped taking their medication, and 2.0% reported that they had stopped staying in care (Table 1).

#### 3.1.5. Recommendations for reducing HIV-related stigma in Ghana

Study participants provided recommendations on what they think various groups in Ghana (community members, health care providers, and adolescents) including themselves should do to help reduce HIV-related stigma. The recommendations are presented below. They can be used for the development of evidence-based HIV destigmatization interventions.

#### 3.1.6. PLWH

There were 246 qualitative responses for what PLWH should do to reduce HIV-related stigma. Using a thematic approach, eight themes emerged from the qualitative analysis: (i) HIV status disclosure to peers, friends and family; (ii) awareness creation and education of the public on HIV; (iii) correct misconceptions; (iv) confidence; (v) legal action and support; (vi) avoid self-stigma, (vii) treatment, and (viii) educate oneself about HIV.

As part of their recommendations, the majority of the study participants proposed that PLWH should avoid disclosing their HIV status to peers, friends, and family (cited 94 times). Others recommended that PLWH need to create awareness and educate the public on HIV (cited 48 times), correct people’s misconceptions related to HIV (cited 36 times), be confident (cited 18 times), seek legal action and support (cited 13 times), avoid self-stigma (cited 14 times), seek treatment (cited 13 times), and educate themselves about HIV (cited 2 times). Some of the things they specifically said were:

***“*** *Never disclose your HIV status to people, especially life partners.”*

*“We must educate the community about certain misconceptions since most of the stigma is centered in the community”.*

*“Fight for our right by taking legal actions.”*

#### 3.1.7. Community level

Regarding what should be done at the community level to reduce HIV-related stigma in Ghana, 64.9% of the study participants said community members should thank PLWH when they disclose their status, 95.5% said ask PLWH how they can be supported, and 95.3% said reassure PLWH that the disclosure of their status will not change the nature of their relationships. Additionally, 96.5% of the study participants stated that community members need to educate themselves about HIV, 94.1% said avoid being judgmental of PLWH, and 95.8% said treat PLWH with dignity and respect (Table 1).

#### 3.1.8. Health care provider level

In response to what health care providers should do to reduce HIV-related stigma in Ghana, majority (96.6%) of the study participants suggested that health care providers should participate in mandatory training on HIV-related stigma led by PLWH, be empathetic to PLWH during treatment and care (95.5%), participate in training on HIV transmission, prevention, and care (95.8%), and identify behaviors that are stigmatizing (95.3%). In addition, study participants (94.3%) stated that health care providers need to avoid showing occupational fear of acquiring HIV, ensure that training in HIV is provided to non-HIV specialty health care providers (95.3%), make HIV-related stigma a priority in HIV care and treatment (94.8%), and develop and enforce policies regarding HIV-related stigma (94.8%) (Table 1).

#### 3.1.9. Adolescent level

When it came to what should be done to reduce HIV-related stigma among adolescents who do not have HIV, responses from participants included educate adolescents on HIV and how it is transmitted (97.0%), educate adolescents on the negative effects of HIV-related stigma (96.8%), and encourage adolescents to talk freely about HIV (92.8%). Regarding what should be done to reduce HIV related stigma among adolescents living with HIV, majority of the study participants (96.5%) said discrimination against adolescents living with HIV should be stopped. Other participants said remind adolescents living with HIV to take their HIV medications (96.0%), and educate school teachers and staff about HIV and how it is transmitted (96.3%). Additionally, 94.3% of the study participants said boarding schools should feed HIV students when they need to eat so they can take their medications on time, and 94.3% said schools should have flexible schedules for adolescents living with HIV so they can keep their clinical appointments.

#### 3.1.10. Caregivers and sex workers

On the issue of what should be done to reduce HIV related stigma among caregivers, recommendation from participants include not to shun HIV caregivers (91.6%), not to make caregivers feel guilty for taking care of someone living with HIV (96.5%), and the need to not talk negatively about HIV caregivers (97.0%). To reduce HIV-related stigma among sex workers, 33.7% of the study participants proposed that sex work should not be criminalized and 89.1% said people should avoid talking negatively about sex workers (Table 1).

#### 3.1.11. Men who have sex with men

Regarding reducing HIV-related stigma among MSM, the study participants said people should not gossip about MSM (23.0%), health care providers should not deny MSM treatment and care (81.4%), and health care providers should treat MSM with respect when they seek care (36.6%).

### 3.2. Bivariate and multivariate analysis

Using cross tabulation, we conducted bivariate analysis of age, sex, and education by the perceptions of PLWH on the factors that cause HIV-related stigma in Ghana, challenges HIV-related stigma poses for HIV treatment and care among PLWH, and what should be done at the community, health care provider, and adolescent levels to reduce HIV related stigma in Ghana.

With regards to the causes of HIV-related stigma in Ghana by age, sex, and education, we found a significant relationship with age (*p*-value = 0.040) (Table 2A). We did not find any significant relationships with the other variables. When it came to the challenges HIV-related stigma poses for treatment and care among PLWH, we again found a significant relationship with age (*p*-value = 0.022) (Table 2B). On what can be done to reduce HIV-related stigma at the community level in Ghana by sex, age and education, there was significant a relationship between education and thanking PLWH for disclosing their status (*p*-value = 0.003) (Table 2C). We also found significant relationships between age and the remaining variables in the domain. Specifically, there were significant relationships between age and reassuring PLWH that the disclosure of their status will not change their relationship (*p*-value = 0.008), educating oneself about HIV (*p*-value = 0.018), avoiding being judgmental (*p*-value = 0.016), treat PLWH with dignity (*p*-value = 0.001), do not deny PLWH employment (*p*-value = 0.015), stop blaming PLWH for getting the disease (*p*-value = 0.011), and provide quality care to PLWH (*p*-value = 0.041).

**Table 2A:** Bivariate analysis of age, sex, and education by causes of HIV-related stigma.

	Age, *n* (%)				Sex, *n* (%)		Level of education, *n* (%)	
Causes of HIV-related stigma in Ghana	18–24	25–40	41+	Male	Female	No school/primary school	Junior high/college/vocational	*p*-value
Ignorance								0.852 (age)
Yes	7 (63.6)	105 (71.4)	172 (70.2)	97 (68.8)	186 (71.3)	95 (68.8)	190 (71.4)	0.605 (sex)
No/I do not know	4 (36.4)	42 (28.6)	73 (29.8)	44 (31.2)	75 (28.7)	43 (31.2)	70 (28.6)	0.588 (edu)
Belief that only certain groups of people get HIV					48 (18.4)	27 (19.6)	51 (19.2)	0.040 (age)*
Yes	5 (45.4)	32 (21.8)	41 (16.7)	29 (20.6)	213 (81.6)	111 (80.4)	215 (80.8)	0.597 (sex)
No/I do not know	6 (54.6)	115 (78.2)	204 (83.3)	112 (79.4)				0.925 (edu)
Fear of contracting HIV								0.231 (age)
Yes	5 (45.4)	51 (34.7)	69 (28.2)	45 (31.9)	78 (29.9)	44 (31.9)	81 (30.4)	0.673 (sex)
No/I do not know	6 (54.6)	96 (65.3)	176 (71.8)	96 (68.1)	183 (70.1)	94 (68.1)	185 (69.6)	0.768 (edu)
Negative comments about people who are tested for HIV								0.503 (age)
Yes	8 (72.7)	107 (73.3)	166 (67.8)	97 (68.8)	183 (70.4)	97 (70.3)	185 (69.8)	0.740 (sex)
I do not know	3 (27.3)	39 (26.7)	79 (32.2)	44 (31.2)	77 (29.6)	41 (29.7)	80 (30.2)	0.921 (edu)
Perception that people deserve to get HIV because of their choices								0.630 (age)
Yes	4 (36.4)	36 (24.5)	58 (23.7)	37 (26.2)	59 (22.6)	37 (26.8)	61 (22.9)	0.415 (sex)
No/I do not know	7 (63.6)	111 (75.5)	187 (76.3)	104 (73.8)	202 (77.4)	101 (73.2)	205 (77.7)	0.388 (edu)

* indicates are statistically significant.

**Table 2B:** Bivariate analysis of sex, age, and education by challenges of HIV-related stigma on treatment and care.

	Age, *n* (%)				Sex, *n* (%)		Level of education, *n* (%)	
Challenges of HIV-related stigma on treatment and care	18–24	25–40	41+	Male	Female	No school/ primary school	Junior high/college/ vocational	*p*-value
Sigma related to HIV has affected your treatment and care in the past 12 months								0.446 (age)
Yes	11 (100)	134.(91.2)	229 (93.5)	134 (95.0)	239 (81.6)	124 (89.9)	251 (94.4)	0.200 (sex)
No/I do not know	0 (0.0)	13 (8.8)	16 (6.5)	7 (5.0)	22 (8.4)	14 (10.1)	15 (5.6)	0.096 (edu)
Which have you done because of HIV-related stigma in the past 12 months (multiple)								
Not taking my medicine								0.925 (age)
Selected								
0 (0.0)	2 (1.4)	3 (1.2)	1 (0.7)	4 (1.5)	1 (0.7)	4 (1.5)	0.661 (sex)	
Not selected	11 (100)	145 (98.6)	242 (98.8)	140 (99.3)	257 (98.5)	137 (99.3)	262 (98.5)	0.665 (edu)
Not staying in care								0.584 (age)
Selected	0 (0.0)	4 (2.7)	4 (1.6)	1 (0.7)	7 (2.7)	2 (1.5)	6 (2.3)	0.270 (sex)
Not selected	11 (100)	143 (97.3)	241 (98.4)	140 (99.3)	254 (97.3)	136 (98.5)	260 (97.7)	0.721 (edu)
Unwilling to disclose my HIV/AIDS status								0.022 (age)*
Selected					258 (98.9)	136 (98.6)	262 (98.5)	
Not selected	10 (90.9) 1 (9.1)	143 (97.3) 4 (2.7)	244 (99.6) 1 (0.4)	138 (97.9) 3 (2.1)	3 (1.1)	2 (1.4)	4 (1.5)	0.427 (sex) 0.667 (edu)

* indicates are statistically significant.

**Table 2C:** Bivariate analysis of sex, age, education by what should be done at the community level to reduce HIV-related stigma in Ghana.

	Age, *n* (%)				Sex, *n* (%)	Level of education, *n* (%)		
What should be done at the community level to reduce HIV-related stigma in Ghana (Multiple)	18–24	25–40	41+	Male	Female	No school/primary school	Junior high/college/ vocational	*p*-value
Thank people living with HIV for disclosing their status								0.030 (age)
Selected	8 (72.7)	83 (56.5)	170 (69.4)	99 (70.2)	162 (62.1)	76 (55.1)	186 (69.9)	0.102 (sex)
Not selected	3 (27.3)	64 (43.5)	75 (30.6)	42 (29.8)	99 (37.9)	62 (44.9)	80 (30.1)	0.003 (edu)*
Ask people living with HIV how they can be supported								0.034 (age)
Selected	9 (81.8)	136 (92.5)	240 (98.0)	132 (93.6)	252 (96.6)	132 (95.7)	254 (95.5)	0.175 (sex)
Not selected	2 (18.2)	11 (7.5)	5 (2.0)	9 (6.4)	9 (3.4)	6 (4.3)	12 (4.5)	0.940(edu)
Reassure people living with HIV that the disclosure of their status will not change your relationship								0.008(age)*
Selected	9 (81.8)	136 (92.5)	239 (97.6)	133 (94.3)	250 (95.8)	133 (96.4)	252 (94.7)	0.511 (sex)
Not selected	2 (18.2)	11 (7.5)	6 (2.4)	8 (5.7)	11 (4.2)	5 (3.6)	14 (5.3)	0.460(edu)
Educate oneself about HIV								0.018(age)*
Selected	9 (81.8)	141 (95.9)	239 (97.6)	134 (95.0)	254 (97.3)	134 (97.1)	256 (96.2)	0.234 (sex)
Not selected	2 (18.2)	6 (4.1)	6 (2.4)	7 (5.0)	7 (2.7)	4 (2.9	10 (3.8)	0.780(edu)
Avoid being judgmental								0.016(age)*
Selected	9 (81.8)	134 (91.2)	236 (96.3)	131 (92.9)	247 (94.6)	132 (95.7)	248 (92.2)	0.485 (sex)
Not selected	2 (18.2)	13 (8.8)	9 (3.7)	10 (7.1)	14 (5.4)	6 (4.3)	18 (6.8)	0.329(edu)
Treat people living with HIV with dignity and respect								0.001(age)*
Selected	9 (81.8)	136 (92.5)	241 (98.4)	132 (93.6)	253 (96.9)	132 (95.7)	255 (95.9)	0.115(sex)
Not selected	2 (18.2)	11 (7.5)	4 (1.6)	9 (6.4)	8 (3.1)	6 (4.3)	11 (4.1)	0.920(edu)
Do not deny people with HIV employment								0.015(age)*
Selected	9 (81.8)	136 (92.5)	238 (97.1)	132 (93.6)	250 (95.8)	134 (97.1)	250 (94.0	0.340 (sex)
Not selected	2 (18.2)	11 (7.5)	7 (2.9)	9 (6.4)	11 (4.2)	4 (2.9)	16 (6.0)	0.171(edu)
Stop blaming people living with HIV for getting the disease								0.011(age)*
Selected	9 (81.8)	137 (93.2)	239 (97.6)	133 (94.3)	251 (96.2)	133 (96.4)	253 (95.1)	0.394 (sex)
Not selected	2 (18.2)	10 (6.8)	6 (2.4)	8 (5.7)	10 (3.8)	5 (3.6)	13 (4.9)	0.559(edu)
Provide quality care to people living with HIV								0.041(age)*
Selected	9 (81.8)	134 (91.2)	235 (95.9)	129 (91.5)	248 (95.0)	129 (93.5)	250 (94.0)	0.162 (sex)
Not selected	2 (18.2)	13 (8.8)	10 (4.1)	12 (8.5)	13 (5.0)	9 (6.5)	16 (6.0)	0. 841(edu)

* indicates are statistically significant.

There were also significant relationships between what should be done by health care providers to reduce HIV-related stigma in Ghana by sex, age, and education. Specifically, Table 2D shows significant relationships between age and the need to be empathetic towards PLWH during treatment and care (*p*-value = 0.030), participation in training on HIV transmission, prevention, and care (*p*-value = 0.003), identifying behaviors that are stigmatizing (*p*-value = 0.008), avoid showing occupational fear of acquiring HIV (*p*-value = 0.018), make HIV stigma a priority in HIV care and treatment (*p*-value = 0.001), and creating and enforcing policies regarding HIV-related stigma (*p*-value = 0.015).

**Table 2D:** Bivariate analysis of sex, age, education by what should be done by health care providers to reduce HIV-related stigma in Ghana.

	Age, *n* (%)				Sex, *n* (%)		Level of education, *n* (%)	
What should be done by health care providers to reduce with HIV related stigma in Ghana (Multiple)	18–24	25–40	41+	Male	Female	No school/primary school	Junior high/college/vocational	*p*-value
Be empathetic towards people living with HIV during treatment and care								0.030(age)*
Selected	8 (72.7)	83 (56.5)	170 (69.4)	99 (70.2)	162 (62.1)	76 (55.1)	186 (69.9)	0.102 (sex)
Not selected	3 (27.3)	64 (43.5)	75 (30.6)	42 (29.8)	99 (37.9)	62 (44.9)	80 (30.1)	0.003(edu)*
Participate in training on HIV transmission, prevention, and care								0.003(age)*
Selected	9 (81.8)	136 (92.5)	240 (98.0)	132 (93.6)	252 (96.6)	132 (95.7)	254 (95.5)	0.175 (sex)
Not selected	2 (18.2)	11 (7.5)	5 (2.0)	9 (6.4)	9 (3.4)	6 (4.3)	12 (4.5)	0.940(edu)
Identify behaviors that are stigmatizing								0.008(age)*
Selected	9 (81.8)	136 (92.5)	239 (97.6)	133 (94.3)	250 (95.8)	133 (96.4)	252 (94.7)	0.516 (sex)
Not selected	2 (18.2)	11 (7.5)	6 (2.4)	8 (5.7)	11 (4.2)	6 (3.6)	14 (5.3)	0.460(edu)
Avoid showing occupational fear of acquiring HIV								0.018(age)*
Selected	9 (81.8)	141 (95.9)	239 (97.6)	134 (95.0)	254 (97.3)	134 (97.1)	256 (96.2)	0.234 (sex)
Not selected	2 (18.2)	6 (4.1)	6 (2.4)	7 (5.0)	7 (2.7)	4 (2.9)	10 (3.8)	0.654(edu)
Provide training in HIV to non-HIV specialty doctors								0.025(age)*
Selected	9 (81.8)	134 (91.2)	236 (96.3)	131 (92.9)	247 (94.6)	132 (95.7)	248 (93.2)	0.485 (sex)
Not selected	2 (18.2)	13 (8.8)	9 (3.7)	10 (7.1)	14 (5.4)	6 (4.3)	18 (6.8)	0.329(edu)
Make HIV stigma a priority in HIV care and treatment								0.001(age)*
Selected	9 (81.8)	136 (92.5)	241 (98.4)	132 (93.6)	253 (96.9)	132 (95.7)	255 (95.9)	0.115 (sex)
Not selected	2 (18.2)	11 (7.5)	4 (1.6)	9 (6.4)	8 (3.1)	6 (4.3)	11 (4.1)	0.920(edu)
Create and enforce policies regarding HIV-related stigma								0.015(age)*
Selected	9 (81.8)	136 (92.5)	238 (97.1)	132 (93.6)	250 (95.8)	134 (97.1)	250 (94.0)	0.340 (sex)
Not selected	2 (18.2)	11 (7.5)	7 (2.9)	9 (6.4)	11 (4.2)	4 (2.9)	16 (6.0)	0.171(edu)
Avoid discriminating against HIV patients								0.011(age)*
Selected	9 (81.8)	137 (93.2)	239 (97.6)	133 (94.3)	251 (96.2)	133 (96.4)	253 (95.1)	0.394 (sex)
Not selected	2 (18.2)	10 (6.8)	6 (2.4)	8 (5.7)	10 (3.8)	5 (3.6)	13 (4.9)	0.559(edu)
Provide quality care to people living with HIV								0.041(age)*
Selected	9 (81.8)	134 (91.2)	235 (95.9)	129 (91.5)	248 (95.0)	129 (93.5)	250 (94.0)	0.162 (sex)
Not selected	2 (18.2)	1398.8)	10 (4.1)	12 (8.5)	13 (5.0)	9 (6.5)	16 (6.0)	0.841(edu)

* indicates are statistically significant.

We further found significant relationships between what should be done to reduce HIV-related stigma among adolescents living with HIV in Ghana by sex, age, and education, with age being the most significant variable. There were also significant relationships between age and stopping discrimination against adolescents living with HIV (*p*-value = 0.007), educating schoolteachers and staff about HIV and how it is transmitted (*p*-value = 0.013), educating students about HIV and how it is transmitted (*p*-value = 0.014), boarding schools should feed HIV students on medication when they need to eat (*p*-value = 0.021), and having flexible school schedule for adolescents living with HIV so students can attend clinical appointments (Table 2E).

**Table 2E:** Bivariate analysis of sex, age, education by what should be done to reduce HIV-related stigma among adolescents living with HIV in Ghana.

	Age, *n* (%)				Sex, *n* (%)		Level of education, *n* (%)	
What should be done to reduce HIV-related stigma in among adolescents living with HIV (Multiple)	18–24	25–40	41+	Male	Female	No school/primary school	Junior high/college/vocational	*p*-value
Stop discrimination against adolescents living with HIV								0.007(age)*
Selected	10 (90.9)	137 (93.2)	242 (98.8)	135 (95.7)	253 (96.9)	132 (95.7)	258 (97.0)	0.534 (sex)
Not selected	1 (9.1)	10 (6.8)	3 (1.2)	6 (4.3)	8 (3.1)	6 (4.3)	8 (3.0)	0.485(edu)
Remind adolescents living with HIV to take their HIV medications								0.008(age)*
Selected	9 (81.8)	139 (94.6)	240 (98.0)	135 (95.7)	252 (96.6)	135 (97.8)	254 (95.5)	0.684 (sex)
Not selected	2 (18.2)	8 (5.4)	5 (2.0)	6 (4.3)	9 (3.4)	3 (2.2)	12 (4.5)	0.239(edu)
Educate school teachers and staff about HIV and how it is transmitted								0.013(age)*
Selected	10 (90.9)	135 (91.8)	240 (98.0)	132 (93.6)	252 (96.6)	132 (95.7)	254 (95.5)	0.175 (sex)
Not selected	1 (9.1)	12 (8.2)	5 (2.0)	9 (6.4)	9 (3.4)	6 (4.3)	12 (4.5)	0.940(edu)
Educate students about HIV and how it is transmitted								0.014(age)*
Selected	9 (81.8)	134 (91.2)	237 (96.7)	130 (92.2)	249 (95.4)	130 (94.2)	251 (94.4)	0.187 (sex)
Not selected	2 (18.2)	13 (8.8)	8 (3.3)	11 (7.8)	12 (4.6)	8 (5.8)	15 (5.6)	0.942(edu)
Boarding schools should feed HIV students on medication when they need to eat								0.021(age)*
Selected	9 (81.8)	137 (93.2)	238 (97.1)	133 (94.3)	250 (95.8)	134 (97.1)	251 (94.4)	0.511 (sex)
Not selected	2 (18.2)	10 (6.8)	7 (2.9)	8 (5.7)	11 (4.2)	4 (2.9)	15 (5.6)	0. 217(edu)
Encourage adolescents living with HIV to take their HIV medications								0.017(age)*
Selected	9 (81.6)	139(94.6)	239(97.6)	132(93.6)	254(97.3)	133(96.4)	255(95.9)	0.070(sex)
Not selected	2(18.2)	8(5.4)	6(2.4)	9(6.4)	7(2.7)	5(3.6)	11(4.1	0.802 (edu)
Have flexible school schedule for adolescents living with HIV so students can attend clinical appointments								0.004(age)*
Selected	9(81.8)	133(90.5)	238(97.1)	131(92.9	248(95.0)	132(95.7)	249(93.6)	0.384 (sex)
Not selected	2(18.2)	14(9.5)	7(2.9)	10(7.1)	13(5.0)	6(4.3	17(6.4)	0.401(edu)

* indicates are statistically significant.

We conducted a multivariate analysis. After adjusting for demographic variables (sex, age, and education) and community recommendations to reduce HIV-related stigma in Ghana, we found that age and education were significant. Study participants aged 26–40 were 53.3% less likely to be thankful to PLWH for disclosing their status compared to participants who were 41 years and above (AOR 0.567, CI 0.368–0874, *p*-value = 0.010) (Table 3). Also, participants with no school or only primary school education, were 48.5% less likely to be thankful compared to participants with junior high or college education (AOR 0.515, CI 0.333–0796, *p*-value = 0.003) (Table 3). When it came to supporting PLWH, after we adjusted for sex, age and education, education was significant. Study participants aged 18–24 years were 91.9% less likely to support PLWH than participants aged 41 years and above (AOR 0.081, CI 0.013–0510, *p*-value = 0.007). Study participants who were 26–40 years were 75% less likely than participants aged 41 years and above to show support for PLWH (AOR 0.250, CI 0.085–0.737, *p*-value = 0.012) (Table 3). We found that study participants who were 18–24years old were 89.7% less likely than those who were 41 years and above to select educating oneself as a means to reduce HIV-related stigma at the community level (AOR 0.103, CI 0.017–0.617, *p*-value = 0.013) (Table 3).

**Table 3:** Multivariate analysis of age, sex, and education by what the community can do to reduce HIV-related stigma in Ghana.

	Adjusted odds ratio	95% Confidence interval (CI)	*p*-value
Thank PLWH for disclosing their status			
Male (Reference: female 1.00)	1.359	0.868–2.129	0.180
18–24 years (Reference: 41 + years 1.00)	0.848	0.209–3.434	0.817
26–40 years	0.567	0.368–0.874	0.010*
No school/primary school (Reference: junior high school-college/voc etc. reference 1.00)	0.515	0.333–0.796	0.003*
Ask PLWH how they can be supported			
Male (Reference: female 1.00)	0.502	0.191–1.319	0.162
18–24 years (Reference: 41 + years 1.00)	0.081	0.013–0.501	0.007*
26–40 years	0.250	0.085–0.737	0.012*
No school/primary school (Reference: junior high school-college/voc etc. reference 1.00)	0.871	0.309–2.456	0.794
Reassure PLWH that relationship will not change			
Male (Reference: female 1.00)	0.728	0.281–1.883	0.512
18–24 years (Reference: 41 + years 1.00)	0.108	0.018–0.632	0.014*
26–40 years	0.307	0.111–0.849	0.023*
No school/primary school (Reference: junior high school-college/voc etc. reference 1.00)	1.304	0.449–3.781	0.626
Educate oneself about HIV			
Male (Reference: female 1.00)	0.527	0.178–1.562	0.248
18–24 years (Reference: 41 + years 1.00)	0.103	0.017–0.617	0.013*
26–40 years	0.577	0.182–1.829	0.350
No school/primary school (Reference: junior high school-college/voc etc. reference 1.00)	1.076	0.321–3.606	0.906
Avoid being judgmental			
Male (Reference: female 1.00)	0.744	0.318–1.743	0.496
18–24 years (Reference: 41 + years 1.00)	0.168	0.031–0.920	0.040*
26–40 years	0.389	0.162–0.935	0.035*
No school/primary school (Reference: junior high school-college/voc etc. reference 1.00)	1.453	0.554–3.808	0.447
Treat PLWH with dignity and respect			
Male (Reference: female 1.00)	0.744	0.318–1.743	0.496
18–24 years (Reference: 41 + years 1.00)	0.168	0.031–0.920	0.040*
26–40 years	0.389	0.162–0.935	0.035*
No school/primary school (Reference: junior high school-college/voc etc. reference 1.00)	1.453	0.554–3.808	0.447
Do not deny PLWH employment			
Male (Reference: female 1.00)	0.653	0.260–1.642	0.365
18–24 years (Reference: 41 + years 1.00)	0.136	0.024–0.778	0.025*
26–40 years	0.358	0.135–0.949	0.039*
No school/primary school (Reference: junior high school-college/voc etc. reference 1.00)	1.914	0.617–5.941	0.261
Stop blaming PLWH for getting the disease			
Male (Reference: female 1.00)	0.656	0.249–1.730	0.395
18–24 years (Reference: 41 + years 1.00)	0.106	0.018–0.623	0.013*
26–40 years	0.338	0.120–0.953	0.040*
No school/primary school (Reference: junior high school-college/voc etc. reference 1.00)	1.186	0.404–3.482	0.756
Provide quality care to PLWH			
Male (Reference:female 1.00)	0.543	0.238–1.238	0.146
18–24 years (Reference: 41 + years 1.00)	0.163	0.030–0.892	0.036*
26–40 years	0.427	0.182–1.003	0.051
No school/primary school (Reference: junior high school-college/voc etc. reference 1.00)	0.804	0.338–1.910	0.621

* indicates are statistically significant.

## 4. Discussion

The study examined PLWH perceptions of the causes of HIV-related stigma in Ghana, challenges that HIV-related stigma poses to treatment and care for PLWH, and recommendations on what can be done to reduce HIV-related stigma among various groups in Ghana (community members, health care providers, and adolescents living with HIV) including PLWH. In so doing, it addressed a gap in the literature around what PLWH believe are the causes of HIV-related stigma in Ghana, how HIV-related stigma impacts their ability to seek treatment and care, and what PLWH recommend various groups in Ghana do to reduce the stigma associated with HIV.

### 4.1. Causes of HIV-related stigma in Ghana

Ignorance is the main cause of HIV-related stigma in Ghana. The majority of PLWH (70.5%) who participated in the study indicated that ignorance is a contributing factor to the stigma associated with HIV in Ghana. This is not uncommon in many parts of sub-Saharan Africa including Ghana where cultural norms, and social pressure cause people to not want to be associated with the disease, let alone learn about it. This is an issue, as knowledge about HIV has been associated with reducing stigma. According to the Centers for Disease Control and Prevention (CDC), the lack of knowledge about HIV causes people to fear the disease, leading to negative judgements about PLWH. (25) In their study on HIV-related stigma and discrimination in Ghana, Tenkorang et al. corroborated the CDC finding. They found that Ghanaian men and women with relatively high knowledge about HIV/AIDS had low stigmatizing and discriminatory attitudes (26).

The majority of study participants who believed that ignorance about HIV was the cause of HIV-related stigma in Ghana were 41 + years. This finding is consistent with a 2019 survey conducted on HIV stigma knowledge, which noted that despite scientific advances and decades of HIV advocacy and education, young adults and adolescents overwhelmingly lack information about the basics of HIV and their HIV status (27). It is therefore not surprising that older people worldwide are more aware of their HIV status compared to younger people. Of the estimated 1.2 million people in the United States (US) living with HIV in 2019, for every 100 people of that population aged 13–24 years, 56 knew their HIV status. Among people aged 25–34 years, 72 knew their status, and among people aged 45 and above, about 92 knew their HIV status (28). This statistic for the younger population needs to be reversed as awareness of one's HIV status is a crucial step to HIV transmission prevention.

### 4.2. Challenges of HIV-related stigma for HIV treatment and care

In our study, we found that age was significantly associated with adherence to HIV treatment and care. Taking HIV medication sometimes requires PLWH to divulge their diagnosis to others, especially when bottles of pills are found in their possession, or when they are seen taking a particular type of pill (29). The stigma associated with taking antiviral medications has the tendency to reduce medication adherence among PLWH. This is consistent with the findings of Golin et al. who reported that younger HIV-infected adults were likely to be nonadherent to medications due to the fear of stigma and potential rejection by friends or family (30). In research conducted in 10 HIV treatment programs in Burundi, Cameroon and the Democratic Republic of Congo, Newman et al. (2012) found that PLWH who were ≥50 years of age had 1.6 times the odds of being adherent to their HIV medications compared to their younger counterparts (people 18–49 years of age) (31). In Spain, Branas et al. (2008) compared rates of ART adherence between PLWH who were ≥65 years of age to PLWH who were less than 65 years. They found that although the difference in adherence was not statistically significant, a greater proportion of PLWH who were ≥65 (70.8%) reported ≥95 percent adherence compared to PLWH who were 65 years of age or younger (58.1%) (32).

### 4.3. Recommendations for reducing HIV-related stigma in Ghana

#### 4.3.1. Community members

Oftentimes, the behavior of distant relatives, friends, and fellow employees in Ghana, contribute to HIV-related stigma among PLWH (33). This is consistent with a 2014 Ghana Development Health Survey finding that showed that very few (22%) study participants expressed positive attitudes towards HIV stigma indicators (34). Additionally, in their study on families and communities living with HIV in Taiwan, Manijsin et al. found that over 25% of young adults were worried about their chances of contracting HIV because they were either living or working in close proximity with PLWH, sharing meals, glasses, or eating meals prepared by PLWH (35). A qualitative study conducted in Liuzhou, China among PLWH found that PLWH who were stigmatized by their family also faced discrimination at work and got fired once their HIV-positive status was known (36). The results of another study conducted in Ghana on HIV-related stigma showed that 12% of the study participants indicated that they would change jobs if someone they worked with became infected with HIV (15). In a cross-sectional survey on perceived discrimination conducted among 451 PLWH and 292 caregivers in Haiti, researchers found that 32% of caretakers and their children experienced discrimination. These situations demonstrate how the rights of PLHW, and their families have been violated due to stigma (37). The negative behavior of community members towards PLWH constitutes a major drawback in the fight against HIV (38). Educating community members on HIV, how it is transmitted, strengthening social support systems, and implementing culturally appropriate educational interventions may help to reduce community-related HIV stigma (4). This is consistent with a previous study conducted by Brown et al., which found that PLWH engagement with community members contributed to generating empathy, dispelling HIV misinformation, and lowering stigma associated with PLWH (39).

Unfortunately, the place PLWH go to seek treatment and care in Ghana, is one of the places where they encounter serious forms of stigmatization and discrimination (40). A cross-sectional study conducted in three hospitals in Cape Coast, Ghana, in 2017, found that health care providers minimized contact with PLWH, denied them care, avoided treating them or isolated PLHW from other patients (41, 42). Reasons for such behavior included the fear of getting infected with HIV in the course of their work, and their negative opinions about PLWH. These are also some of the reasons participants in our study mentioned (41). The same cross-sectional study also found that nurses were more likely than medical doctors to exhibit stigmatizing behavior (41). A study conducted in five African countries found higher levels of HIV-related stigma among nurses towards PLWH. This is worrying, given the fact that nurses are usually the frontline staff who provide health care services to HIV patients (43). While Ghana has made some strides to control HIV, the gains may be eroded if HIV-related stigma among health care providers is not addressed.

#### 4.3.2. Adolescents living with HIV

HIV prevalence among adolescents and young people is an issue of ongoing global concern. According to the United Nations Children's Fund (UNICEF), adolescents represent a growing share of PLWH worldwide (44), and even though AIDS-related deaths are declining among all other age groups, deaths among adolescents have increased over the past decade (44). This may be due to a generation of perinatally-HIV infected children who are now growing into adolescence, or the lack of knowledge about the disease and how it is transmitted.

School attendance is critical to the lives and development of adolescents, including those living with HIV (45). Public day and boarding schools are the two common forms of academic settings for adolescents in many countries, including Ghana (46). In those environments, teachers, school staff, and peers become their main community and source of support as daily contact with families and caregivers is limited (47). However, this very environment poses challenges to adolescents living with HIV as most of their peers and staff are ignorant about HIV and thus, engage in stigmatizing behaviors. Such behaviors make it difficult for adolescents living with HIV to disclose their HIV status, take their medications, or even access health care facilities compared to their counterparts who attend day schools, as policies in boarding schools restrict independence and control access to healthcare facilities and external providers. These circumstances serve as barriers to accessing daily antiretroviral treatment (ART), cause self and experienced stigma, and provide limited privacy and confidentiality. A qualitative study from Kisumu, Kenya reported that the fear of stigma prevented HIV status disclosure to school staff among adolescents living with HIV and led to higher rates of disengagement from care.

The stigma associated with HIV also has been shown to be associated with specific psychological challenges such as depression and anxiety for young people living with HIV, as well as decreased self-esteem (48). These psychological experiences have been associated with increased rates of sexual and substance use risk behaviors (49), as well as decreased adherence to ART, and medical appointments. Given the range of negative psychosocial and medical outcomes that adolescents and young adults living with HIV experience due to HIV-related stigma, it is important to equip this population with skills to combat negative societal influences. It is also important to educate adolescents, schoolteachers, and staff, and to get schools to provide school-based support for adolescents living with HIV so they can optimize their health and wellbeing.

#### 4.3.3. Limitation of the study

A limitation of the study is the fact that data was collected from only one administrative region of Ghana. As a result, findings cannot be generalized to the entire population of Ghana. This is a limitation because some other regions like the Northern and Upper administrative regions of Ghana are more rural and have a completely different cultural and social context with regards to HIV-related stigma compared to the Ashanti region. Irrespective of this, the data collected represents the voice and perspectives of PLWH and HIV-related stigma in the Ashanti region. Another limitation of the study is the fact that we did not examine the relationship between religion and HIV-related stigma seeing that some of the shaming and blaming behaviors mentioned in the paper appear to emanate from religious worldviews.

## 5. Conclusion

Eliminating HIV-related stigma is an important and necessary contribution to the global fight against HIV. To address HIV-related stigma in Ghana so as to increase HIV testing uptake, disclosure of HIV status, adherence to treatment, and to improve upon the quality of life of PLWH, it is important for countries, including Ghana, to focus on PLWH and to engage them in determining strategies for HIV stigma reduction at several levels simultaneously. This is because, interventions targeted at potential sources of HIV-related stigma have a greater probability of success.

## Data Availability

The original contributions presented in the study are included in the article/Supplementary Material, further inquiries can be directed to the corresponding author.
